# The Role of STATs in Ovarian Cancer: Exploring Their Potential for Therapy

**DOI:** 10.3390/cancers15092485

**Published:** 2023-04-26

**Authors:** David Standing, Emma Feess, Satvik Kodiyalam, Michael Kuehn, Zachary Hamel, Jaimie Johnson, Sufi Mary Thomas, Shrikant Anant

**Affiliations:** 1 Department of Cancer Biology, University of Kansas Medical Center, Kansas City, KS 66103, USA; 2 Department of Otolaryngology, University of Kansas Medical Center, Kansas City, KS 66160, USA

**Keywords:** STAT, signaling, isoforms, immune cell, targeted therapy

## Abstract

**Simple Summary:**

Ovarian cancer remains the deadliest cancer of the female reproductive system. There is a significant body of evidence that has demonstrated that Signal Transducers and Activators of Transcription (STATs) play a critical role in ovarian cancer progression. STAT proteins are highly activated in ovarian cancer and are thus attractive targets for developing targeted therapeutic strategies. This review highlights the current knowledge of STAT biology in ovarian cancer, with an emphasis on epithelial ovarian cancer, while also providing a comprehensive discussion on STAT specific inhibitors in preclinical and clinical stages of development. As a result, this review will be relevant to a wide readership, encompassing aspects of biology and therapeutics.

**Abstract:**

Ovarian cancer (OvCa) is a deadly gynecologic malignancy that presents many clinical challenges due to late-stage diagnoses and the development of acquired resistance to standard-of-care treatment protocols. There is an increasing body of evidence suggesting that STATs may play a critical role in OvCa progression, resistance, and disease recurrence, and thus we sought to compile a comprehensive review to summarize the current state of knowledge on the topic. We have examined peer reviewed literature to delineate the role of STATs in both cancer cells and cells within the tumor microenvironment. In addition to summarizing the current knowledge of STAT biology in OvCa, we have also examined the capacity of small molecule inhibitor development to target specific STATs and progress toward clinical applications. From our research, the best studied and targeted factors are STAT3 and STAT5, which has resulted in the development of several inhibitors that are under current evaluation in clinical trials. There remain gaps in understanding the role of STAT1, STAT2, STAT4, and STAT6, due to limited reports in the current literature; as such, further studies to establish their implications in OvCa are necessitated. Moreover, due to the deficiency in our understanding of these STATs, selective inhibitors also remain elusive, and therefore present opportunities for discovery.

## 1. Introduction

Ovarian cancer (OvCa) remains the deadliest gynecological malignancy, with a 5-year survival rate across all subtypes of <50% [1]. Epithelial OvCa (EOC). The most common amongst these subtypes, accounting for 85–90% of cases, will be the focus of the studies presented in this review. Though there have been advancements with surgical procedures and chemotherapy, particularly with the approval of PARP inhibitors, which have contributed to improvements in overall survival (OS), 5- and 10-year relative survival rates for advanced disease (stage III and stage IV) remain dishearteningly low [2]. Relative survival for stage III and IV disease at 5 years is ~35% and ~23%, respectively [2]. Moreover, OvCa is frequently diagnosed at a late stage due in part to a lack of preventive screening and symptom presentation at early stages [3]. The poor survival statistics indicate that current standard-of-care procedures involving cytoreductive surgery in conjunction with chemotherapy are insufficient to treat advanced disease; therefore, identifying gaps in our knowledge of OvCa biology is essential to improving patient response. 

**Figure 1:**
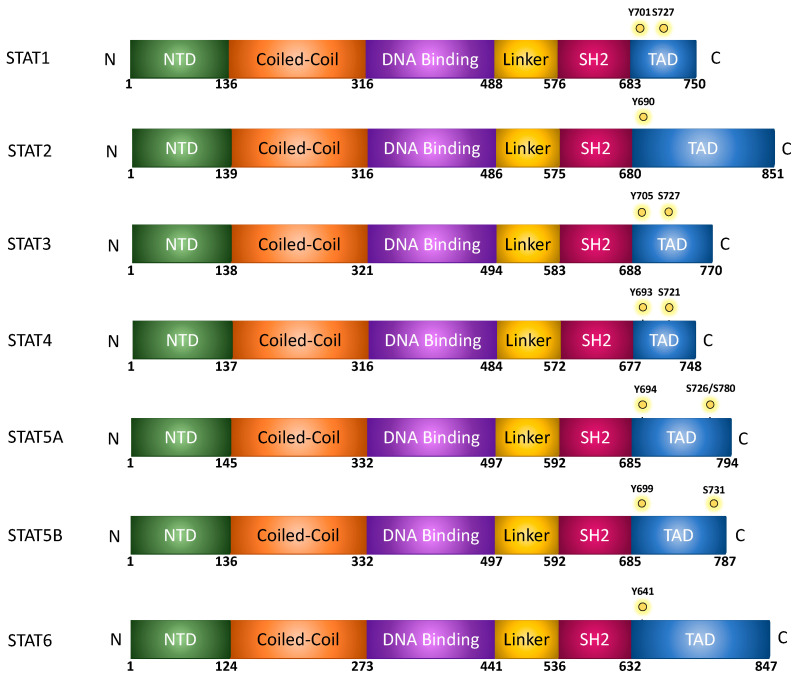
Protein/domain structure of STAT family members.

There is an increasing body of evidence demonstrating the importance of Signal Transducers and Activators of Transcription (STATs) in OvCa, demonstrating that STATs promote cellular growth, stemness, metastasis, and chemoresistance of cancer cells [4,5,6,7,8]. The STAT family is comprised of seven distinct proteins, STAT1, STAT2, STAT3, STAT4, STAT5A and 5B, and STAT6, which will be discussed in detail below [9]. Briefly, the structure among STAT proteins remains highly conserved, with all STAT proteins encoding a coiled-coil (CC) domain, a DNA-binding (DB) domain, and a SRC homology 2 (SH2) domain. The SH2 domain is separated from the DB domain by a linker domain (LD) (Figure 1) [10]. Following activation by a variety of receptors and proteins of the Janus kinase (JAK) family, phosphorylated STAT proteins translocate into the nucleus and bind to target DNA sequences, promoting gene transcription [11].

**Figure 2:**
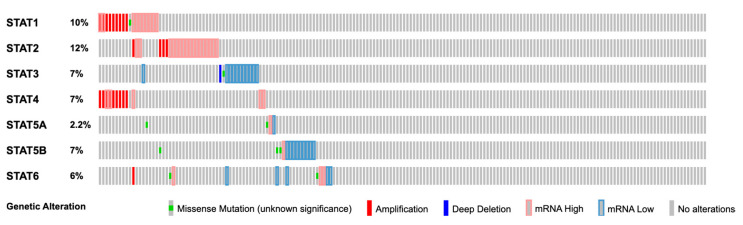
Mutation profile of STAT proteins in ovarian serous cystadenocarcinoma. Oncoprint of mutations of TCGA, Firehose Legacy data from 182 samples of ovarian serous cystadenocarcinoma.

As shown in Figure 2, the mutation profile amongst the various STAT proteins demonstrates a 2–12% mutational rate from 182 samples of ovarian serous cystadenocarcinoma, the most common subtype of OvCa. Interestingly, STAT1 and STAT4 mutations appear as co-occurrences and are mostly comprised of gene amplifications. Despite these observations, mutation rates are low compared to other common mutations associated with EOC, such as P53 (~87%) and MYC (42%). This would suggest that the involvement of STATs in OvCa would be dependent on other factors, such as the sustained activation of the signaling pathway by various activating factors described in later sections.

Understanding the interaction between the tumor microenvironment (TME) and cancer cells has become increasingly critical to improving therapeutic response. The TME is composed of several components such as blood vessels, extracellular matrix, fibroblasts, and immune cell populations, which have been shown to closely interact with tumor cells and modulate tumorigenesis, progression, metastasis, and chemoresistance [12]. There is accumulating evidence that targeting the components of the TME can profoundly influence patient response to treatment [13]. In the context of EOC, protumor functions of immune cells found in malignant ascites are common. Aberrant molecular signaling and crosstalk between the stroma and the tumor facilitate tumor progression and response to therapy. A key transcription factor often upregulated in OvCa, STAT3, and other STAT proteins contributes to the protumorigenic functions and drives the TME towards a tumor supporting niche [14]. For example, OvCa tumor cells have been demonstrated to secrete exosomes containing miR-222-3p that polarize macrophages towards an M2 lineage via a SOCS3/STAT3 mechanism [15]. STAT6 has also been shown to drive M2 polarization by modulating Trim24, a CREB-binding protein-associated E3 ligase that curtails M2 derivation [16]. Foxp3+CD4+ regulatory T cells (Tregs) suppress T-cell activation, resulting in tumor growth and progression. Considered to be a master transcription factor controlling Treg function, Foxp3 is directly regulated by STAT3 and STAT5 [17]. IL-2 and TGF-β, which are common factors found in the TME, induce STAT3 binding to the FOXP3 gene, which has been demonstrated through in vivo studies [18,19,20]. High levels of Tregs have been shown to correlate with a reduction in anti-tumor lymphocytes in the TME in addition to poor patient survival [21,22,23]. From these examples, it is clear that STATs play a critical role in the TME, further potentiating tumor progression. In this comprehensive review, we will discuss the functional significance of STAT proteins in OvCa, with an emphasis on EOC, by exploring their roles in cancer cells and other cells in the TME, and we will address potential therapeutic implications. 

## 2. Structure of STATs

STAT proteins are highly conserved, with the molecular weights of full-length STAT proteins being around 80–100 kDa [24]. As described in Figure 1, at the N-terminus, STATs contain an N-terminal domain (NTD), which is followed by a coiled-coil (CC) domain. The DNA-binding (DB) domain is located near the center of the protein and is separated from the SH2 domain by a linker (LD). At the c-terminus of the protein, there is a trans-activating domain (TAD) [10]. The NTD has importance in STAT protein interactions, including STAT dimers, transcription factors, cofactors, and receptors [25]. The CC domain, which encodes four-helix bundles is involved in protein–protein interactions that control nuclear import and export processes [25]. Moreover, while the DB domain is important for binding the chromatin and inducing transcription, it also encodes sequences that are essential for nuclear import. Moreover, in addition to providing structural organization, the linker domain plays a role in transcriptional regulation of STAT1 [26]. The SH2 domain primarily functions in recognizing phosphotyrosine motifs within cytokine receptors. Additionally, upon phosphorylation of a tyrosine residue by a tyrosine kinase such as Janus kinase, the SH2 domain acts as a point for dimerization of two STAT proteins, either a heterodimer or homodimer [27]. Once STAT proteins dimerize, they move into the nucleus where they bind the gamma activated sequences (GAS) to initiate gene transcription. The TAD encodes a conserved serine phosphorylation site through which the protein interacts with co-factors/activators of transcription. In addition, the domain regulates protein stability, especially in STATs 4, 5, and 6; but not STAT1, 2, and 3, wherein sequences can be targeted by the ubiquitin–proteosome degradation pathway [25,28]. 

### STAT Alternative Isoforms

**Figure 3:**
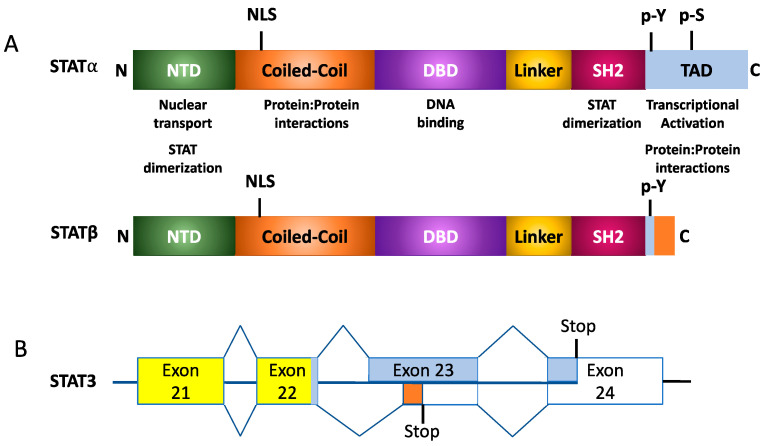
Structural organization of STAT isoforms. (**A**) Generalized protein organization of alpha and beta STAT isoforms following alternative splicing. (**B**) Example of splicing for STAT3α and STAT3β isoforms.

In addition to full-length STATs, also termed STATα, alternative splicing and proteosomal cleavage results in the formation of STATβ and STATγ isoforms, respectively, as shown in Figure 3 [24]. STATβ protein possesses a modified 55 amino acid C-terminal that lacks the TAD. This impacts the protein’s ability to facilitate target gene transcription [29,30]. Studies have also demonstrated that overexpression of STATβ proteins may result in the protein having a dominant negative function. However, under physiological conditions, the two isoforms may also encode unique functions; for example, with STAT3, mice that only express the Stat3α isoform appear to have a defective response to endotoxic shock [31]. On the other hand, while STAT3 knockout is embryonic lethal, this can be rescued by expressing the STAT3β isoform [32]. STAT4 also has two isoforms, a full-length Stat4α or a truncated Stat4β lacking 44 amino acids in the C-terminus. IL-12 is a key cytokine that activates the expression of these two isoforms of STAT4. Microarray-based studies demonstrated that while both STAT4 isoforms activate 98 genes, Stat4α and Stat4β uniquely activated 32 and 29 genes, respectively [33]. What this suggests is that the β-isoforms of STAT3 and STAT4 possess transcription-inducing activity, further suggesting that these isoforms do not just possess a dominant-negative function. This further suggests that STAT-mediated signaling in response to cytokine activation is coordinated between the variants of the protein. Another method of regulating STAT proteins occurs in conditions without alternative splicing, but rather through proteolytic cleavage of the C-terminus. This eliminates the TAD, resulting in the STATβ isoform. Although STAT3, STAT5a, STAT5b, and STAT6 proteins form the γ-isoform, they each do so in different contexts. Located within the nucleus of myeloid progenitor cells, proteases cleave STAT5a and STAT5b to induce STAT5γ formation. This process is not affected by the tyrosine-phosphorylation state of STAT5 [24]. Just as with the cleavage of STAT5, in myeloid progenitor cells, proteases modify the C-terminal of STAT3α. However, this protease only cleaves the phosphorylated form of the full-length STAT3α. This phosphorylation requirement is a key difference between Stat3α and STAT5a/b. Such proteolytic processes occur primarily due to serine proteases, though calpain also demonstrates an affinity for cleaving STAT3 and STAT5 in platelets while cleaving STAT6 in mast cells [24].

## 3. STAT Activation and Signaling

**Figure 4:**
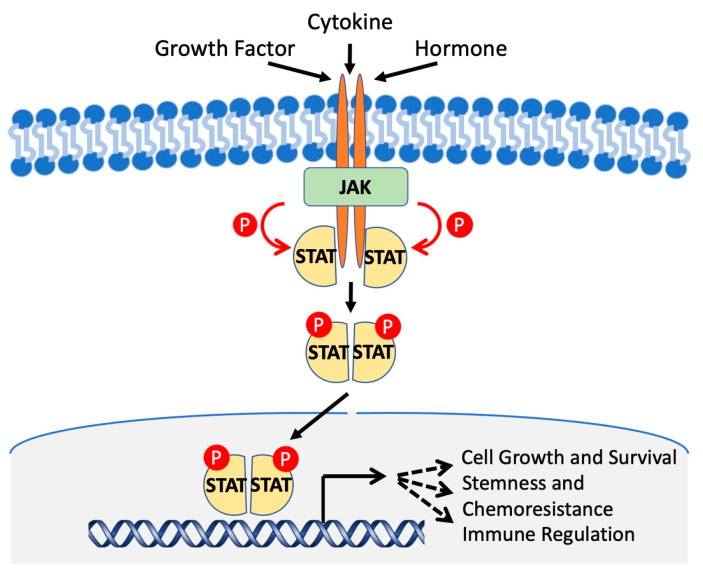
STAT signaling. Cytokines, growth factors, and hormones bind to numerous receptors that result in the recruitment of JAK proteins, which, in turn, phosphorylate STAT proteins. Phosphorylated STATs sequester away from the receptor complex, dimerize, and translocate to the nucleus to enhance downstream target gene transcription.

In the cytoplasm, STAT proteins exist in their inactive form before phosphorylation occurs, resulting in an active dimer that can translocate into the nucleus and bind to target DNA sequences and activate transcription [11]. A variety of signaling molecules and kinases are responsible for the activation of STAT proteins, but the primary and most well-known is the JAK/STAT pathway (Figure 4). Canonical signaling via JAK, and non-canonical activation of STATs are explored in detail in a recent review by Hu et al. [10]. Briefly, Janus kinase (JAK) is often activated by a transmembrane cytokine receptor [26]. The type of JAK and subsequent STAT activated depends on the cytokine and the receptor it activates, summarized in Table 1. Class I cytokine receptors respond to a wide range of cytokines including IL-6 and IL-2 that activate STAT3, STAT5, and STAT6. Interferon-γ activates STAT1, while interferon types II and III activate both STAT1 and STAT2. IL-10 cytokine activates STAT3 [34,35]. All four Janus kinases (JAK)—JAK1, JAK2, JAK3, and TYK2 (tyrosine kinase 2)—are activated by autophosphorylation after binding to the transmembrane receptor. STATs are then activated by phosphorylation by JAKs [36]. Canonical activation via JAK-induced stimuli results in phosphorylation of tyrosine residues within the TAD domain, promoting STAT dimerization, and nuclear import. STATs can also be activated at serine residues within the TAD, though this phosphorylation event remains inconclusive regarding function. Several studies have demonstrated that phosphorylation at these serine residues can enhance transcriptional activity, potentiating the effects of tyrosine phosphorylation [37,38,39]; however, it has also been reported to suppress transcriptional activity, further demonstrating the complexity of this signaling pathway [40,41,42]. Moreover, serine phosphorylation may therefore contribute to modulating transcriptional activity in response to select activators, regulating transcription of select target genes. These phosphorylation sites are summarized in Figure 1. There are a variety of other kinases that do not require a receptor and can activate STAT proteins without JAK. SRC is an example of a kinase that does not require a receptor [43]. Activation of STAT allows translocation from the cytoplasm into the nucleus, where it activates several genes related to development and survival of the cell [44]. 

## 4. The Effect of STATs on OvCa Cells

### 4.1. STAT1

STAT1 mediates a response to type I, II, and III interferons, which results in either inflammatory or apoptotic responses [11]. STAT1 is an important transcription factor in bone growth and formation [57]. In addition, it has been shown to have anti-tumor effects through signaling in mesenchymal stromal cells (MSCs), although this occurs following the switching of STAT1 homodimers to STAT1:STAT2 heterodimers in response to interferon-α (IFNα) [58]. STAT1 has also been implicated in OvCa development and therapeutic response. However, this is debatable due to conflicting evidence that demonstrates a dual role for STAT1 both as a poor and favorable prognostic factor. It has been demonstrated to be overexpressed in high-grade serous ovarian cancer (HGSOC) compared to normal ovarian tissue, and yet this higher expression was correlated with longer overall survival [59]. On the other hand, there is evidence demonstrating that STAT1 promotes EOC proliferation by inducing the expression of nitric oxide synthase (iNOS) [60,61,62]. Interestingly, the induction of nitric oxide (NO) can also induce cellular apoptosis by increasing p53 expression [63]. It has also been shown that integrin β1 (ITGB1) is upregulated in EOC and promotes proliferation of tumor cells by inducing STAT1 expression. STAT1 has also been identified to promote cellular adhesion, migration, and invasion in several cancers, including OvCa. Tian et al. demonstrated that STAT1 induced EOC cell invasion and migration by inhibiting TGF-β signaling [64]. Paradoxically, STAT1 has also been shown to inhibit these phenotypes by enhancing transcription of CXCL9, CXCL10, and CXCL11, leading to longer overall survival and progression-free survival in patients, and reduction of ascites formation and tumor burden in ID8 OvCa murine models of disease [65,66]. Collectively, these data demonstrate a dual role for STAT1 in OvCa; therefore, further studies are required to characterize its function more decisively.

### 4.2. STAT2

STAT2 is activated by Type I and Type III interferons [34]. Activation of STAT2 results in the formation of a complex with STAT1 and IRF9 (Interferon Regulatory Factor 9) [11]. It plays a role in defending against viral infections [67]. The role of STAT2 in OvCa remains largely unknown. In a single report by Wang et al., the authors demonstrate that fibrillin-1 (FBN1) is significantly expressed in cisplatin-resistant HGSOC cells and promotes the resistance through a FBN1/VEGFR2/STAT2 signaling axis [7]. RNA-sequencing of cisplatin-resistant cells identified that resistance was associated with angiogenesis and glucose metabolism based on gene ontology analyses [7]. Moreover, GSEA analysis indicated that FBN family members were significantly upregulated in cisplatin-resistant HGSOC [7]. Mechanistically, FBN1 knockout decreased glycolysis and angiogenesis, which further resulted in increased cisplatin sensitivity [7]. Moreover, following immunoprecipitation (IP) of FBN1 coupled with mass spectrometry (MS), VEGFR2, was determined as a key molecule that binds FBN1 [7]. FBN1 was found to induce phosphorylation of VEGFR2 at Tyr1054, which resulted in downstream activation of focal adhesion kinase (FAK) and AKT signaling pathways [7]. This, in turn, induced phosphorylation of STAT2 at Tyr690, promoting nuclear translocation. Collectively, these studies demonstrate that FBN1 activates STAT2 to modulate glycolysis and angiogenesis in HGSOC to promote cisplatin resistance. This demonstrates an important role for STAT2 in HGSOC that may have potential for therapeutic interventions. At the very least, further studies are warranted to determine the molecular functions of STAT2 in OvCa.

### 4.3. STAT3

STAT3 is activated by a variety of cytokines, including IL-6 and IL-10 [68]. It is important for immunosuppression, which leads to its strong association as a promoter of certain cancers, including OvCa [69]. STAT3 is also important for the proliferation of neutrophils during infection in emergency granulopoiesis [70]. More importantly, STAT3 has been connected to induced chemo- and radioresistance in various tumor types [71,72,73,74]. The role of STAT3 in OvCa has been extensively studied with a significant body of evidence demonstrating the importance of STAT3 in tumor development, survival mechanisms, metastasis, stemness, and chemoresistance. Conversely, STAT3 inhibition suppresses these phenotypes and improves survival, suggesting potential therapeutic implications, which will be discussed in greater detail in later sections. In a recent publication by Gao et al., the authors summarize via a systematic review and meta-analysis the impact of STAT3 and phospho-STAT3 expression on clinicopathology and prognosis of OvCa [14]. Briefly, the authors describe 16 studies that encompassed 1747 OvCa patients and summarized STAT3/pSTAT3 expression in OvCa samples relative to normal ovarian tissue, as well as their impact on overall survival (OS) and progression-free survival (PFS). The meta-analysis described data from patients of a variety of histological subtypes, including serous, clear cell, endometrioid, and mucinous, as well as undifferentiated tumors [14]. The authors concluded that both STAT3 and pSTAT3 are upregulated in OvCa compared to normal, and that expression was correlated with FIGO stage, tumor grade, and poorer prognosis regarding both OS and PFS [14]. In the following sections, we have compiled a narrative outlining the impact of STAT3 on OvCa cells.

#### 4.3.1. Proliferation and Survival

As a transcription factor, the persistent activation and translocation of STAT3 into the nucleus promotes transcription of genes such as c-Myc, cyclin D1, Bcl-2, Bcl-xL, and survivin to drive cell growth and survival. Huang et al. determined that STAT3 is highly activated in EOC cell lines compared to non-cancerous ovarian surface epithelium [5]. Mechanistically, cell lines expressing highly phosphorylated species of STAT3 exhibited elevated expression of downstream mediators, cyclin D1, and Bcl-xL compared to cells with low phosphorylated STAT3 [5]. When cells are subjected to hypoxic conditions, STAT3 phosphorylation is significantly induced; however, STAT3 knockdown reduced proliferation of A2780 endometrioid/clear cell carcinoma cells [75]. More recently, it was determined that STAT3 activation increased oncogenic miR-216a, which directly inhibited the tumor suppressor phosphatase and tensin homolog (PTEN), promoting tumor cell growth and resistance [76]. In a recent study by Li et al., methylenetetrahydrofolate dehydrogenase 2 (MTHFD2) was found to promote cell growth and tumor aggressiveness via activation of STAT3 [77]. Similarly, Wang et al. determined that tripartite motif 47 (TRIM47) enhanced OvCa proliferation by activating STAT3 signaling [78]. Moreover, TRIM47 knockdown suppressed STAT3 phosphorylation, which resulted in decreased expression of Myc mRNA and abrogated cell growth and survival in both clear cell carcinoma SKOV3 and HGSOC OVCAR3 cell lines [78]. To confirm that TRIM47 activity was dependent on STAT3, the authors overexpressed TRIM47 in SKOV3 cells, and proceeded to ectopically suppress STAT3 expression using siRNA. TRIM47 overexpression induced cellular proliferation; however, in the setting of STAT3 downregulation, the protein was unable to potentiate cell growth confirming STAT3 dependency [78]. 

#### 4.3.2. Angiogenesis

Significant outgrowth of cancer cells ultimately necessitates access to increased blood supply. STAT3 also is significantly involved in angiogenesis, with several reports providing mechanistic insights. For example, following vascular endothelial growth factor (VEGF) release by cancer cells, new blood vessels are formed via a STAT3 signaling axis. In particular, activated STAT3 has been demonstrated to co-localize with VEGF in tissues of EOC [79]. STAT3 has also been well established as a signaling mediator of IL-6 stimulation that directly induces transcription of VEGF, as well as activates HIF1α, which is also a potent transcription factor controlling VEGF expression [80,81]. Recently, Yin et al. reported that Wip1 controlled tumor metastasis and platinum resistance via a STAT3/VEGF dependent mechanism [82]. Specifically, overexpression of Wip1 in the clear cell SKOV3 OvCa cells resulted in downregulation of FGF-16, VEGF, and thrombospondin-1 [82]. Moreover, in endometroid/clear cell A2780 and low grade serous HeyA8 OvCa cells transduced with Wip1 targeting shRNA, VEGF expressing increased compared to vector control, demonstrating a direct link between Wip1 and VEGF [82]. Phenotypically, Wip1 overexpression decreased tube formation of HUVEC cells in vitro and CD31 expression in peritoneal tumors of mice injected with SKOV3 Wip1 OE cells compared to vector control [82]. Molecularly, Wip1 overexpression decreased VEGF mRNA and protein expression, as well as STAT3 phosphorylation [82]. Conversely, Wip1 knockdown resulted in increased VEGF expression and STAT3 phosphorylation, which was ameliorated following treatment with the STAT3 inhibitor, Stattic [82]. In another study by Martincuks et al., resistance to the PARPi Olaparib was associated with the activation of STAT3 signaling, resulting in the increased expression of target genes cyclin D1, VEGF, Mcl1, and Bcl-xL [83]. Moreover, ectopic silencing of STAT3 by siRNA suppressed these genes and phenotypically resulted in impaired tumor cell proliferation and re-sensitization towards Olaparib [83]. Collectively, there is evidence of a STAT3/VEGF activation loop that perpetuates OvCa progression through the support of vascular development within the tumor microenvironment.

#### 4.3.3. Metastasis

Typically, the formation of new blood vessels is accompanied by the increased dissemination of cancer cells from the primary tumor site. However, OvCa metastasis differs from that of the majority of solid tumors, i.e., it takes a transcoelomic route instead of utilizing blood vessels or lymphatics. The role of STAT3 in cancer metastasis, including in OvCa, has been extensively characterized. Epithelial-to-mesenchymal transition (EMT) is one method utilized by cancer cells to enhance metastatic potential [84]. In OvCa, STAT3 inhibition is associated with the concomitant reduction in N-cadherin, Vimentin, and TWIST1, critical genes in the EMT process [4,85,86]. In a study by Zheng et al., it was shown that AKT2 promoted OvCa progression through a Pyruvate kinase M2 (PKM2)-STAT3/NF-κB mechanism [87]. Specifically, the authors identified that AKT2 was upregulated in OvCa cell lines compared to immortalized non-cancerous ovarian surface epithelial cells [87]. PKM2 is a critical enzyme in glycolysis, and is related to tumorigenesis, progression, and metastasis. The authors determined that ectopic knockdown of AKT2, but not AKT1 or AKT3, decreased PKM2 expression in both clear cell SKOV3 and HGSOC HeyA8 cells [87]. Conversely, overexpression of AKT2 increased expression of PKM2 in both OvCa cell lines, demonstrating a direct relationship [87]. Phenotypically, PKM2 overexpression significantly increased cell migration and invasion, which was impaired following shRNA-mediated silencing of AKT2 [87]. As proof of principle, the authors further demonstrated that AKT2 inhibition impaired PKM2-mediated formation of lung metastases in a tail vein colonization murine mouse model [87]. Molecularly, the authors identified that PKM2 overexpression induced both STAT3 and NF-κB expression, but not AKT2 [87]. Meanwhile, knockdown of AKT2 suppressed PKM2, STAT3, and NF-κB expression in both SKOV3 and HeyA8 cells [87]. Collectively, these data demonstrate that AKT2 is an upstream activator of PKM2, which subsequently activates STAT3 and NF-κB signaling to promote OvCa metastasis. In a recent study by Chong et al., the authors investigated the role of HSP90 in EMT [86]. Specifically, the authors identified that HSP90 cooperated with STAT3 to regulate TWIST1 levels in cell lines from multiple cancer types, including OvCa. In treating cells with the HSP90 inhibitor, 17-AAG, the authors observed a decrease in migration in the wound healing assay [86]. There was also a reduction in TWIST1 mRNA and promoter activity [86]. To understand the mechanism of 17-AAG-mediated suppression of TWIST1, the authors interrogated the expression of known HSP90 client proteins that have been reported to regulate TWIST1, which includes STAT3. Using proximity ligation assay to visualize HSP90 interactions with client proteins, they observed that HSP90 interactions with STAT3 were significantly reduced following incubation with 17-AAG [86]. The authors also performed a ChIP assay to assess HSP90 inhibition on STAT3 transcriptional activity. A2780 and SKOV3 cells treated with 17-AAG exhibited significantly reduced STAT3 binding within the TWIST1 promoter, suggesting that HSP90 enhances the activity and binding of STAT3 [86]. Co-immunoprecipitation assays further confirmed that 17-AAG treatment impaired STAT3:HSP90 interactions [86]. Together, these data demonstrate that HSP90 enhances the direct STAT3 regulation of TWIST1 transcription.

#### 4.3.4. Stemness

The concept of cancer stem cells (CSCs) has gained much interest with respect to its implications in chemoresistance and tumor recurrence. Though the markers of CSCs remain debatable, the stemness phenotype has been more decisively characterized by the potential of self-renewal and differentiation, as well as resistance to apoptotic mechanisms [88]. There is accumulating evidence that STAT3 is a significant regulator of the stemness phenotype in a variety of cancers, including OvCa. Specifically, STAT3 has been identified in CSC-enriched spheroid cultures and correlates with putative CSC markers ALDH1A1, β-catenin, c-myc, CD24, and Nanog [89,90,91]. More recently, it was shown by Giordano et al. that the L1 cell adhesion molecule (L1CAM) promotes HGSOC stemness and progression via FGFR1/Src/STAT3 signaling [92]. L1CAM has been previously demonstrated to enhance HGSOC growth, metastasis, and chemoresistance, with little insight regarding CSCs. Hence, the authors sought to define the role of L1CAM in CSCs and determined that L1CAM is essential and adequate for HGSOC tumorigenesis, self-renewal, and chemoresistance [92]. Mechanistically, spheroid forming efficiency (SFE) was decreased following L1CAM knockdown in OVCAR3 cells [92]. Conversely, overexpression of L1CAM in Ov90 cells significantly enhanced SFE, suggesting a role for L1CAM in the maintenance of HGSOC CSCs [92]. Moreover, L1CAM overexpression significantly enhanced tumor forming ability of SKOV3 cells when injected subcutaneously in NOD/SCID/IL2Rγ-null mice at a density of 250 cells/mouse [92]. The authors also evaluated L1CAM expression on paclitaxel sensitivity and determined that Ov90 cells were significantly more resistant to paclitaxel insult when L1CAM was overexpressed compared to mock controls [92]. To delineate the mechanism of L1CAM function, Ov90 cells were subjected to transcriptomic profiling, which identified STAT3 signaling as a highly activated modulator in CSC populations and L1CAM overexpressing cells [92]. The authors validated these findings and further demonstrated that STAT3 was required for L1CAM driven spheroid formation, in which Ov90 cells either expressing L1CAM or a mock vector, were treated with the STAT3 inhibitor Napabucasin and paclitaxel, alone or in combination. Spheroid forming efficiency was significantly impaired following Napabucasin treatment [92]. Finally, the activation of STAT3 by L1CAM was mediated through Src and JAK, as phosphorylation of STAT3 was inhibited following treatment with inhibitors for either of these proteins, even in L1CAM-overexpressing cells [92]. Collectively, these data demonstrate that L1CAM induces stemness and chemoresistance in HGSOC cells via STAT3.

### 4.4. STAT4

STAT4 is activated by IL-12 and IL-23. STAT 4 plays a role in inducing inflammation as well as the differentiation of T helper 1 cells; however, little is known about STAT4’s role in cancer [93]. There is increasing evidence providing insight to the functional role of STAT4 in OvCa, though further studies are necessary to decisively characterize the protein’s function. Zhao et al. determined that STAT4 was overexpressed in EOC and was correlated with poor prognoses by promoting metastatic behavior [94]. Conversely, knockdown of STAT4 reduced omental spread and tumor growth in xenograft murine mouse models, and further extended survival following IP injection of HO-8910 OvCa tumor cells [94]. Mechanistically, the authors demonstrated that STAT4 induced epithelial-mesenchymal transition (EMT) through cancer–stroma interactions, as STAT4 overexpression induced EMT of tumor cells in vivo but failed to induce EMT in vitro [94]. Specifically, STAT4 overexpression was correlated with an increased abundance of CAFs within the tumor microenvironment that was further accompanied by increased concentrations of CAF-secreted factors IL6, CXCL12, and VEGFA [94]. Moreover, conditioned media from HO-8910 cells activated CAFs by inducing Wnt signaling via Wnt7a. In turn, the activated CAFs reciprocally induced EMT of EOC cells, potentiating cellular migration and invasion [94]. 

Building off the aforementioned studies, Li et al. sought to further elucidate the underlying mechanisms of STAT4-mediated EMT [95]. In these studies, the authors identified a relationship between hypoxia, STAT4, and EMT. Under hypoxic conditions, expression of HIF1α, E-cadherin, Vimentin, and STAT4 were increased [95]. In cells that were transduced with a STAT4 expression vector, Vimentin and E-cadherin levels were significantly elevated, and this was accompanied by increased migration of OvCa cells as determined by both scratch plate and Boyden chamber assays [95]. Interestingly, endogenous STAT4 mRNA expression was unaffected by hypoxia, indicating a post-transcriptional regulation of STAT4. Subsequently, the authors identified that miR-200a interacted with sequences within the 3′UTR of STAT4 mRNA [95]. Cells transfected with a miR-200a-mimic demonstrated decreased STAT4 protein expression [95]. Moreover, miR-200a expression was reduced significantly under hypoxic conditions [95]. The authors further confirmed that ectopic overexpression of miR-200a significantly reduced migration and invasion, which was reversed when STAT4 was ectopically co-expressed. Collectively, these studies demonstrate that miR-200a is a negative regulator of STAT4, and that under hypoxic conditions, miR-200a is downregulated, resulting in STAT4 upregulation to drive EMT and the metastasis of OvCa [95]. 

### 4.5. STAT5

STAT5 refers to two different proteins, STAT5a and STAT5b, that are 90% identical, even though they are expressed from two independent genes. Both STAT5a and STAT5b can be activated by IL-3, prolactin, and IL-2 [96,97]. The primary function of STAT5a and STAT5b is regulation of growth, development, and the immune system [98]. STAT5a and STAT5b are also thought to be oncogenic in some cancers, including EOC [99,100]. Though STAT5a/b has been extensively studied in a variety of cancers, its characterization in OvCa remains largely unclear. There are studies suggesting roles for the protein isoforms in cell survival, proliferation, and angiogenesis. However, additional studies are necessary to better delineate how each isoform regulates OvCa progression. In one such study, to examine the causal mechanism of drug resistance in EOC, the use of capillary isoelectric focusing (CIEF) coupled with nano-reversed-phase liquid chromatography (RPLC) identified RELA and STAT5 as major factors associated with carboplatin resistance in EOC tissues [101]. Mechanistically, both RELA and STAT5B directly bound to the promoter of Bcl-xL, enhancing transcription and pro-survival signaling [101]. Moreover, inhibition of RELA and STAT5B using small molecule inhibitors suppressed Bcl-xL expression and resensitized chemoresistant EOC cells to carboplatin insult [101]. In another study by Xu et al., the adipose-derived leptin hormone was observed to promote EOC proliferation by inducing STAT5 phosphorylation in A2780 and SKOV3 cells [102]. The underlying mechanism of leptin-induced cellular proliferation was mediated by the upregulation of miRNAs targeting the 3′UTR of FOXO3, specifically, miR-182 and miR-96 [102]. Leptin exerted its biological activity by inducing phosphorylation of STAT5 via the Leptin receptor, Ob-R [102]. Moreover, siRNA-mediated suppression of STAT5 decreased leptin-induced expression of miR-182 and miR-96, demonstrating that STAT5 directly regulated both miR-182 and miR-96 expression, which could then suppress FOXO3 activity, resulting in enhanced OvCa cell growth [102]. In a study by Chen et al., the authors sought to delineate the role of prolactin in HGSOC tumorigenesis through the regulation of wild type BRCA1 function [103]. Prolactin receptor (PRLR) typically signals through a STAT3/STAT5 signaling axis involving JAK proteins. In this study, the authors observed that prolactin induced the phosphorylation of STAT5, which resulted in the formation of a protein complex between STAT5 and BRCA1 [103]. Interestingly, this interaction did not interfere with nuclear translocation and binding within the p21 promoter, though activation of transcription was significantly impaired [103]. This indicates that under prolactin stimulation, STAT5 binds to BRCA1 to induce a dominant-negative phenotype that inhibits a critical tumor-suppressive function of BRCA1 through the suppression of p21 transcription [103]. Lastly, in a recent publication by Lee et al., the EGFR inhibitor, poziotinib, was found to inhibit OvCa CSCs via HER4 inhibition [104]. The authors isolated CSC-enriched side population cells from the A2780 OvCa cell line and proceeded to assess poziotinib on spheroid forming efficiency. Poziotinib reduced sphere formation by more than 90% compared to controls [104]. Similar results were obtained in SKOV3 cells. Moreover, Poziotinib also reduced cell populations sorted for putative CSC markers, ALDH, CD117, and CD133 [104]. Protein expression of ALDH, CD133, Oct4, Nanog, and KLF4 was also reduced in cells treated with poziotinib, suggesting that the pan-EGFR inhibitor significantly suppressed OvCa CSC properties [104]. Mechanistically, the authors determined that HER4 was the highest among EGFR family genes expressed in OvCa CSC populations, which had been previously shown to regulate STAT5, Akt, and Erk signaling pathways. Therefore, the authors treated OvCa CSCs with poziotinib and determined that phosphorylation of STAT5, Akt, and Erk were significantly decreased [104]. Taken together, these data demonstrate that inhibition of STAT5/Akt/Erk signaling suppresses OvCa CSC viability and growth. Given the insight these studies have collectively provided in understanding the role of STAT5 in EOC, further studies are warranted to further dissect STAT5’s function and mechanisms of activation in OvCa. 

### 4.6. STAT6

Similar to other STAT proteins, STAT6 is also activated by cytokines such as IL-4 and IL-13 [105]. The primary function of STAT6 is in the M2 differentiation of macrophages [106]. In addition, STAT6 plays a role in the T helper 2 response as well as IgE (Immunoglobulin E) production [107]. Like STAT2 and STAT4, the role of STAT6 remains largely unknown, though there is some evidence that STAT6 is associated with supporting the cancer stem cell niche in OvCa. In a single publication by Ruan et al., it was shown that OCT4 was upregulated in the side population (SP) of A2780 and SKOV3 OvCa cells, which increased phosphorylation of JAK and STAT proteins, particularly JAK1 and STAT6 [108]. The authors demonstrated that genetic manipulation of OCT4 expression by either shRNA-mediated knockdown or a lentiviral expression vector that drives overexpression of OCT4, modulated chemoresistance and cellular growth of A2780 and SKOV3 OvCa cells both in vitro and in vivo [108]. Mechanistically, the authors determined that loss of OCT4 resulted in significant suppression of JAK and STAT phosphorylation, particularly JAK1 and STAT6 [108]. Moreover, immunocytochemistry identified subcellular localization of STAT6 to the nucleus following overexpression of OCT4 [108]. As proof of concept, the authors treated SKOV3 and A2780 cells with peficitinib, a JAK inhibitor, to suppress activation of JAK/STAT signaling. The authors further determined that OCT4 mediated inhibition of cellular apoptosis and drug resistance, and invasion was abrogated following treatment with peficitinib, suggesting that JAK/STAT signaling was critical to the OCT4 mechanism of action [108]. These data suggest a role for STAT6 in promoting OvCa progression and resistance, supporting the call for further studies to conclusively establish STAT6’s function in OvCa.

## 5. The Role of STATs in the TME

### 5.1. Macrophages

Understanding how STAT proteins interact with the TME would provide unique insights on the establishment and maintenance of protumor environments. STAT3 has been shown to play a key role in macrophage function within the TME, as significantly increased STAT3 expression indicates an increase of protumor macrophages and poor patient prognosis [109]. Several studies show that an increase of M2 macrophage levels within the TME indicates worse progression-free survival (PFS) and overall survival (OS) in patients with OvCa [110,111,112]. The protumor function of M2 macrophages is due to their immunosuppressive and angiogenic activity by secretion of IL-10 and VEGF, respectively, which allows for cancer cell survival and metastasis to the peritoneal cavity of OvCa [113,114,115]. Several secreted factors contribute to the polarization of macrophages towards a protumorigenic, M2-like phenotype. IL-4, IL-13, IL-10, IL-6, and colony stimulation factor 1 (CSF-1) all contribute towards M2 polarization of tumor-associated macrophages (TAMs) [116,117,118]. The subtype of M2 macrophages, M2d, demonstrates a strong association with protumor TAMs for their angiogenic and immunosuppressive properties, which leads to tumor expansion [119]. The expression of CD163 and CD206, as well as secretion of IL-10, IL6, CCL18, and CCL22, mark the immunosuppressive M2 macrophages [120,121,122]. Production of IL-10 maintains activation of STAT3 in the monocyte cell line, THP-1 [123]. Ablation of STAT3 shows the restoration of antigen-presenting cell (APC) function and the inflammatory activity of macrophages associated with M1 type macrophages and the innate immune system. Restoring the inflammatory immune function of macrophages results in the macrophages acting as tumor suppressers [124]. STAT3 expression is shown to have many downstream effects in macrophages within the TME that increase cancer cell survival.

### 5.2. Dendritic Cells

STAT3 also plays a role in the behavior of dendritic cells within the TME. Dendritic cells function as APCs, providing communication between the innate immune system and the adaptive immune system. These myeloid-derived immune cells present antigens recognized by pattern recognition receptors (PRR) to B cells and T cells of the adaptive immune system, while also producing cytokines such as IL-12, IL-6, and Type I interferons [125]. It has been shown that STAT3 activation alters the function of dendritic cells that are present in the TME. Several studies suggest that the secretion of STAT3 activators, such as IL-6, IL-10, and G-CSF in the TME, prevent DC maturation [126,127,128]. Phosphorylated STAT3 activates IL-6 production, which allows for the sustained autocrine signaling of immature DCs [129]. On the other hand, ablation of STAT3 induces DC maturation and allows for antigen presentation to cytotoxic T lymphocytes (CTLs) [130]. As such, overexpression of STAT3 in DC leads to pro-tumor effects within the TME. 

### 5.3. MDSC

Myeloid-derived suppressor cells (MDSCs) are immunosuppressive cells derived from bone marrow and are often observed at high levels in the TME. MDSCs are known to suppress both CD4+ and CD8+ T cells [131,132,133]. The immunosuppressive quality of MDSCs causes its association with worse patient prognosis when expressed at high levels in the TME [134]. STAT3 has been shown to induce the transcription of proteins related to immunosuppressive MDSCs. In mice studies, S100A9, a target of STAT3, has been shown to prevent the differentiation of myeloid cells into dendritic cells and macrophages, while maintaining the accumulation of MDSCs in the TME [135].

### 5.4. Tregs

Regulatory T cells (Tregs) are CD4+CD25+ T cells that play a role in the immune suppression and regulation of autoreactive T cells [136]. Another marker for Tregs is expression of FOXP3, a master transcription factor specific for Tregs, which is mediated by STAT3 and STAT5 [17]. IL-2 and TGF-β induce STAT3 binding to the FOXP3 gene, which has been demonstrated through in vivo studies [18,19,20]. When Tregs are present at high levels in the tumor microenvironment, there is a reduction in anti-tumor lymphocytes. This results in poor patient survival [21,22,23]. Production of IL-10 and TGF-β is shown to be responsible for the inhibition of CD8+ effector T cell function [137]. Tregs suppress the immune system’s anti-tumor response in a STAT3- and STAT5-mediated fashion. 

## 6. STAT Inhibitors

In this section, we will highlight many inhibitors that are currently on the market to target various STATs, with an emphasis on OvCa, though we have extended our review to additional malignancies to broaden readership. Several inhibitors have been identified for STAT1, STAT5, and STAT6, but the majority have been developed against STAT3, which will encompass the majority of the content in this section. Currently, there is a significant deficiency in specific STAT2 and STAT4 inhibitors; therefore, they will not be discussed in the following sections. Furthermore, there is an array of inhibitors that have been developed commercially for research use; however, our focus will be towards small molecule inhibitors demonstrating direct STAT inhibition and therapeutic implications in cancer. We will explore inhibitors both at preclinical and clinical developmental stages.

### 6.1. STAT1

Fludarabine is a purine analog that inhibits DNA synthesis and selectively inhibits activation of STAT1 [138]. It is widely used in the treatment of chronic lymphocytic leukemia (CLL), non-Hodgkin lymphoma, acute myeloid leukemia (AML),hairy cell leukemia, and OvCa [138,139]. With recent advancements in immunotherapy, fludarabine has regained interest for its ability to modulate immune activity as a suppressive agent and extend the survival of adoptively transferred CD8 T cells in patients [140]. In the context of OvCa, fludarabine has been shown to reduce EOC migration and cell adhesion by inhibiting the FAK/STAT1 signaling pathway [141]. Moreover, fludarabine has also been shown to inhibit VEGF by modulating HIF-1α and PI3K/AKT signaling pathways, resulting in attenuated EOC growth [142]. It has also been shown to have synergistic activity with the standard-of-care agent, cisplatin [143]. More recently, fludarabine is currently being tested in a clinical trial as an immunosuppressant for NK cell therapy in OvCa patients [144]. Specifically, the authors of this Phase I safety trial are seeking to evaluate the safety and toxicity of IP infusion of ex vivo-expanded NK cells for patients with recurrent OvCa [144]. NK cells will be generated from CD34+ hematopoietic progenitor cells isolated from allogeneic umbilical cord blood. The study is currently enrolling patients to be placed in one of two treatment arms: (1) single IP infusion of ex vivo expanded NK cells; (2) single IP infusion of ex vivo expanded NK cells with a non-myeloablative immunosuppressive conditioning regimen with fludarabine and cyclophosphamide [144]. The estimated primary completion date is set for Spring of 2023, and study conclusion for Fall. 

ISS840 is a peptidomimetic inhibitor that selectively inhibits STAT1 and STAT3 homodimerization, with a 20-fold higher specificity for STAT1 [145]. The authors developed ISS840 via modification of a previous STAT3 peptidomimetic inhibitor, ISS610 [145]. In an attempt to improve potency of ISS610, the modifications imparted unexpected specificity towards STAT1 instead of STAT3. Specifically, IC50 values for STAT3-DNA-binding disruption for ISS610 and ISS840 were 42 ± 23 μM and 560 ± 100 μM, respectively [145]. Moreover, IC50 values for STAT1-DNA-binding disruption were 310 ± 145 μM and 31 ± 22 for ISS610 and ISS840, demonstrating a 20-fold higher selectivity of ISS840 for STAT1 versus STAT3 [145]. 

Pravastatin is a non-peptide small molecule that inhibits STAT1 phosphorylation in response to cytokine-induced pathways activation. Specifically, in a study by Zhou et al., pravastatin attenuated IFN-γ induced phosphorylation of STAT1 [146]. IFN-γ was established as a potent activator of STAT1, leading to phosphorylation at Tyr701 and nuclear translocation to induce downstream target gene expression [147]. Using mouse models of aortic atherosclerosis, Zhou et al. further showed that pravastatin-treated mice exhibited reduced atherosclerotic lesion formation, and significantly reduced expression of pSTAT1 and IFN-gamma within the aorta [146]. In the context of cancer, pravastatin has been investigated as an anti-tumor agent in colorectal cancer. In a study by Yeh et al., the authors observed that pravastatin inhibited tumor growth by inducing apolipoprotein A1 (ApoA1) [148]. As a well-established modulator of cholesterol, it is unclear from these studies whether the anti-cancer activity is associated with STAT1 modulation. Therefore, further studies are required to delineate the mechanism of action. In a recent meta-analysis studying the relationship between statin use and OvCa risk, it was found that long-term pravastatin use was associated with higher risk [149]. Again, it is unclear as to the molecular mechanisms driving these results, and whether this is due to modulation of STAT1. Hence, further studies are warranted to assess the role of STAT1 specifically.

As noted previously, the role of STAT1 in cancer remains controversial. However, in a recent study by Chou et al., a novel STAT1 inhibitor was identified using in silico screening tools and was found to suppress CSC populations, stemness properties, and angiogenesis of colorectal cancer cells [150]. Specifically, the authors determined that STAT1 is upregulated in CRC tumor tissues isolated from human subjects and azoxymethane (AOM)/dextran sodium sulfate (DSS)-induced CRC mouse models [150]. Moreover, knockout of STAT1 in CRC cells resulted in significantly reduced tumor growth [150]. As proof of concept, the authors sought to suppress STAT1 activity using pharmacologic approaches. Combing a high-throughput virtual screening platform with the SWEETLEAD chemical database, the authors identified 4′,5,7-trihydroxyisoflavone (THIF) [150]. THIF is abundantly found in soybeans, and shares structural moieties from previously identified STAT1 inhibitors, fludarabine, ISS840, and pravastatin [150]. The authors determined that THIF directly interacted with STAT1 by SPR analysis and further inhibited STAT1 phosphorylation [150]. Treatment of HCT116 CRC cells with THIF further inhibited spheroid growth, a surrogate of stemness, and CSC related genes CD44, CD133, and CD166 [150]. Moreover, THIF suppressed blood vessel formation via CAM assay and angiogenic factors VEGFA, IGFBP6, IGF2, GDNF, and SCF [150]. Previously, the authors identified that Δ9-THC, the primary active ingredient in synthetic cannabis drugs, promotes angiogenesis and CRC progression via specific activation of STAT1 [151]. Interestingly, the authors demonstrate that THIF fails to suppress tumor growth alone yet attenuates Δ9-THC-induced tumor formation in an AOM/DSS-CRC mouse model [150]. Follow-up studies are warranted to further elucidate mechanism of action, as the data suggest that in vitro, genetic, and pharmacologic inhibition of STAT1 reduces stemness and angiogenesis; however, this is not replicated in vivo, except in the presence of a STAT1 activator such as Δ9-THC. 

### 6.2. STAT3

STAT3 has been shown to have profound effects on tumor progression through its effects on the immune system and the tumor itself. STAT3 and its activator, JAK2, have been targeted as treatment options for multiple malignancies. Both natural and synthetic inhibitors have been identified and modulate STAT3 signaling by either direct inhibition of STAT3 or indirectly by inhibiting JAKs or their receptors.

#### 6.2.1. Natural Compounds

Indirubin, an active ingredient of traditional Chinese medicine, has been investigated as a possible therapeutic agent for treating OvCa. A recent study by Chen et al. showed that treatment of OVCAR3 cells with indirubin resulted in a down regulation in phosphorylated-STAT3 at Tyr705. The downregulation of STAT3 was associated with a decrease in cell proliferation and an increase in the pro-apoptotic factors, Bax and cleaved caspase 3, leading to apoptotic cell death [152,153]. Another study using indirubin in a xenograft human prostate model showed a decrease in STAT3 signaling mediated by VEGFR2. This pathway of inhibition suggests that indirubin can inhibit angiogenesis of the tumor by suppressing the JAK2/STAT3 pathway [154]. 

Resveratrol, a stilbenoid commonly found in various berries, grapes, and peanuts, has shown anti-cancer activity in multiple cancer types by modulating the STAT3 signaling pathway [155,156,157,158,159,160]. In the context of OvCa, a study by Zhong et al. determined that resveratrol inhibited the growth of OVCAR3 and CAOV3 cells, inducing G1 phase arrest. This was accompanied by significant suppression of Wnt, Notch, and STAT3 signaling pathways [160]. To elucidate which of the pathways was critical to the observed anti-tumor activity, the authors further treated HGSOC cells with selective Wnt, Notch, and STAT3 inhibitors. Results were recapitulated only with the use of the JAK-specific inhibitor AG490, indicating that resveratrol anti-tumor activity was likely related largely to the inhibition of STAT3 signaling [160]. In a recent publication by Cheuk et al., resveratrol was found to reverse macrophage polarization, resulting in enhanced cisplatin sensitivity [161]. In this study, the authors evaluated the efficacy of resveratrol as a monotherapy or in combination with cisplatin to treat cisplatin resistant breast cancers (BrCa) [161]. Specifically, the authors determined that resveratrol inhibited BrCa by inhibiting IL-6 and STAT3 activation, while also promoting M1 macrophage polarization, increasing the M1:M2 ratio [161]. 

Curcumin, the principal curcuminoid found in turmeric, has been shown to inhibit STAT3 activity in multiple cancer types, including lung, renal, gastric, melanoma, pancreatic, ovarian, prostate, colorectal, and breast cancers, resulting in attenuated cell growth both in vitro and in vivo [162,163,164,165,166,167,168,169,170]. A recent study by Ham et al. investigated the role of STAT3 in CAF-induced chemoresistance in gastric cancer (GC) [170]. The authors determined that conditioned media (CM) isolated from CAFs promoted resistance to 5-fluorouracil (5-FU) [170]. This was mediated by an induction of STAT3 signaling in GC cell lines. Treatment of GC cells with curcumin attenuated CM mediated activation of STAT3 and 5-FU resistance, demonstrating the role of STAT3 and the suitability of using natural products to overcome chemoresistance [170]. In another recent study by Sandhiutami et al., the development of a curcumin nanoparticle was found to potentiate cisplatin efficacy by inhibiting PI3K/AKT and JAK/STAT3 signaling pathways in OvCa [171].

Given curcumin’s poor bioavailability, analogs have been developed which have ultimately been shown to effectively inhibit STAT3 activity [172,173]. For example, the curcumin analogs FLLL31 and FLLL32 demonstrate a potent ability to inhibit STAT3 phosphorylation and thus suppress tumor growth in breast cancer by interfering with the DNA-binding domain of STAT3 and initiating apoptosis [174]. FLLL32 has also been shown to limit DNA-binding activity and thus induce apoptosis in osteosarcoma cell lines [175]. FLLL62, a more soluble analog for FLLL32, has specificity to STAT3 and promotes apoptosis in renal cell carcinoma and melanoma cell lines [164]. As such, these compounds show promise as potential drugs for treating certain cancers through the inhibition of STAT3, though more studies are needed to determine its effectiveness in treating OvCa. HO-3867, HO-4200, and H-4318 were also developed from the curcumin backbone and have shown potent inhibition of STAT3 signaling [176,177,178,179]. These analogs have been studied in OvCa, showing cytotoxic effects by engaging with the DNA-binding domain of STAT3. Collectively, these data demonstrate the feasibility and validity of targeting STAT3 for OvCa.

Corosolic acid (CA) is a pentacyclic triterpene acid found in the ornamental plant, Lagerstroemia speciose [180]. CA has been reported as a STAT3 inhibitor in OvCa, glioblastoma, and osteosarcoma [181]. In the context of OvCa, CA enhanced paclitaxel, cisplatin, and doxorubicin activity by inhibiting STAT3 signaling, while also suppressing the interaction of protumorigenic TAMs and OvCa cells [181]. In a recent publication by Li et al., the authors identified a novel use for CA as a delivery vehicle for anti-cancer agents [182]. The authors determined that CA possesses properties similar to cholesterol and can form cholesterol-free liposomes (CALP) [182]. Interestingly, CALPs exhibit greater membrane fusion and uptake, while also exuding established CA inhibitory activity of STAT3 [182]. When used as a functional delivery vehicle for doxorubicin, there was greater cellular uptake and spheroid penetration, which improved anti-cancer activity compared to doxorubicin-loaded cholesterol-based liposomes [182]. These data demonstrate a novel use for CA that has dual functions for cancer treatment by serving as a modulator of STAT3 signaling and as a delivery system for other chemotherapeutic drugs.

Cucurbitacin (-I, -B, -E) have also been demonstrated to modulate STAT3 signaling by targeting JAK2 and STAT3 [183,184,185]. In the context of OvCa, cucurbitacin-E was found to suppress clear cell carcinoma ES-2 cell proliferation and induce apoptosis, which was accompanied by decreased expression of STAT3 [186]. More recently, cucurbitacin-I was found to inhibit STAT3 but to enhance STAT1 signaling in A549 lung adenocarcinoma cells [187]. Specifically, the authors determined that cucurbitacin-I decreased phosphorylation of STAT3 while inducing that of STAT1 via actin filament disruption [187]. In another recent study by Li et al., cucurbitacin-I was found to induce apoptosis of SKOV3 OvCa cells by modulating oxidative stress [188]. In this study, the authors determined that cucurbitacin-I induced the apoptosis of OvCa cells in a dose- and time-dependent manner, caused by significant increases in reactive oxygen species (ROS) [188]. This was mediated by the downregulation of antioxidant-related genes, Kelch-like ECH-associated protein 1 (KEAP1), and nuclear factor erythroid-derived-2-like 2 (NFE2L2) [188]. Interestingly, crosstalk between STAT3, KEAP1, and NFE2L2 have been published [189,190]. The study by Li et al. does not reference the involvement of STAT3; however, further studies are warranted to dissect a potential connection between STAT3, KEAP1, and NFE2L2 in the suppression of OvCa cell viability. 

Two compounds, named 323-1 and 323-2, are natural chiral isomers of delavatine A, found in the plant Incarvillea delavayi. They have recently been reported as STAT3 inhibitors [191]. Specifically, both stereoisomers directly interact with the SH2 domain within STAT3, thereby suppressing phosphorylation and protein dimerization [191]. Fluorescence polarization confirmed SH2 binding, as SH2-binding peptide GpYLPQTV association was inhibited in their presence [191]. Moreover, IL-6 stimulated phosphorylation of STAT3 at Tyr705 was attenuated in LNCaP cells in the presence of these stereoisomers, while STAT1 phosphorylation in response to IFNγ stimulation was unaffected [191]. Collectively, these data demonstrate that delavatine A stereoisomers 323-1 and 323-2 are potent and selective inhibitors of STAT3 activity.

#### 6.2.2. Synthetic Inhibitors

Stattic is a small molecule that has a high affinity for the SH2 domain of STAT3, thereby inhibiting STAT3 dimerization and function [192,193]. Preclinical studies with Stattic showed that there was a STAT3 blockade resulting in inhibition of stemness, angiogenesis, metastasis, therapy resistance, and tumor growth [194,195,196,197]. For example, in a recent study by Méndez-Clemente et al., the authors demonstrate that Stattic and Tocilizumab, an IL-6R targeting mAb, inhibit prostate cancer growth, migration, and invasion as monotherapies and in combination [196]. Treatment decreased expression of IL-6, CXCL8, VEGF, and vimentin [196]. Ectopic addition of IL-6 rescued cancer cell migration, proliferation, and invasion when Stattic was used as a monotherapy; however, combination with tocilizumab maintained inhibitory activity, even under high dose IL-6 stimulation [196]. In another study published by Guo et al., suppression of STAT3 phosphorylation by Stattic inhibited pancreatic cancer growth and induced mitochondria-mediated apoptosis [194]. Overexpression of STAT3 partially rescued cell growth and decreased apoptosis, indicating that the antitumor effects of Stattic were dependent on STAT3 modulation [194]. Though several studies have confirmed STAT3 blockade by Stattic, recent studies have identified evidence of STAT3 independent effects, raising questions about compound selectivity. In a report by Poria et al., the authors found that in STAT3-deficient prostate cancer cells, Stattic alone had prominent effects on the epigenetic landscape by modulating histone acetylation. Stattic inhibited CCL20 and CCL2, and activated transcription of TNFα, CEBPD, SOX2, and MYC [198]. In another study by Xia et al., Stattic was determined to inhibit glutathione reductase (GSR) resulting in anti-cancer activity in cervical cancer [199]. Specifically, the authors determined that GSR was elevated in cervical cancer tissues [180]. Treatment with Stattic inhibited GSR activity, resulting in cell death [199]. GSH and N-acetyl cysteine were able to reverse these effects, indicating a ROS-dependent mechanism [199]. Surprisingly, inhibition of STAT3 using other pharmacological or genetic approaches failed to induce cell death, suggesting Stattic had STAT3-independent activity [199]. These data provide a new perspective and raise concerns about Stattic as a specific STAT3 inhibitor. 

STA-21 and its structural analogs, LLL-3 and LLL-12, have shown selectivity for the SH2 domain of STAT3, inhibiting STAT3 dimerization and resulting in suppressed glioblastoma, BCR-ABL leukemias, OvCa, medulloblastoma, and breast cancer cell growth [200,201,202,203,204,205,206,207]. Recently, in a study by Vegeli et al., STAT3 activation was linked to oncogenic signaling in hypopharyngeal cells (HCs) following exposure to acidic bile [208]. Suppression of STAT3 by siRNA or STA-21-inhibited acidic bile induced the phosphorylation of HCs [208]. Moreover, STAT3 silencing suppressed activation of inflammatory signaling profiles that included IL6, RELA, WNT5a, and TNFα, suggesting that STAT3 inhibition may serve as a preventive strategy against bile induced hypopharyngeal carcinogenesis [208]. Several studies have recently evaluated STAT3 suppression in OvCa, medulloblastoma, and BrCa using LLL-12 [205,206,207,209]. Specifically, Zhang et al. demonstrated that LLL-12 inhibited STAT3 activity in A2780, SKOV3, CAOV3 and OVCAR5 OvCa cell lines, and when used in combination with cisplatin or paclitaxel, it enhanced the cytotoxicity of these standard-of-care agents, suggesting potential therapeutic implications for EOC [209]. In a follow up study, Zhang et al., developed a novel LLL-12 analog (LLL-12B) using advanced multiple ligand simultaneous docking (AMLSD) [207]. The authors confirmed that LLL-12B interacted with the SH2 domain of STAT3, inhibiting dimerization, which was accompanied by reduced phosphorylation at Tyr705 [207]. LLL-12B suppressed EOC growth and further sensitized cancer cells to paclitaxel and cisplatin [207]. In another study, Chen et al. confirmed similar results in medulloblastoma cells, and further inhibited IL-6-induced STAT3 phosphorylation but not INFγ-induced phosphorylation of STAT1, further demonstrating selectivity of LLL-12B for STAT3 [206]. Lastly, LLL-12B was shown by Pan et al. to inhibit triple-negative BrCa (TNBC) via a mammary fat pad tumor model, supporting the notion of targeting STAT3 for TNBC [205].

There are current studies in several cancer types that use STAT3 decoy oligonucleotides as a potential therapeutic for cancers with upregulated STAT3. Early studies of STAT3 decoy oligonucleotides were conducted in head and neck squamous cell carcinoma (HNSCC) [210]. The decoy was found to bind to activated STAT3 and inhibit activity by blocking its binding to its cognate DNA recognition element. Moreover, daily administration of the decoy inhibited HNSCC growth in a xenograft model [211]. Given that the decoy did not demonstrate any toxicity in a non-human primate model, the compound was moved to the clinic with a First-in-human phase 0 trial [212,213]. No dose-limiting toxicities were observed in the trial, and there were reduced STAT3 levels in the tumors. Systemic administration showed inhibition in the size of tumor, and decreased STAT3 target genes [213]. Efforts continue to be made in the discovery of effective ways to deliver oligonucleotide (ODN) decoys. Cationic small lipid nanoparticles (SLN) hold potential as a delivery mechanism for STAT3 decoys due to their stability, low cytotoxicity, and minimal immunogenicity [214,215]. The effects of SLN ODN decoys in OvCa have been investigated in vivo using SKOV3 xenograft mouse models. The use of SLN to deliver a STAT3 decoy showed an increase in tumor apoptosis through upregulated Bax and cleaved caspase 3 [216,217]. SLN STAT3 decoy also showed a higher rate of tumor apoptosis than STAT3 that was not delivered in a nanoparticle [217]. These studies show that STAT3 decoys induce anti-tumor effects when injected intratumorally; however, the inability to deliver these decoys systemically is still a challenge that needs to be considered. 

LC28 was developed by Huang et al. as a novel STAT3 inhibitor that interacted with the DNA-binding domain [218]. Using structural moieties of other known STAT3-DNA-binding domain inhibitors and electrostatic-based drug design, the authors were able to synthesize LC28 and 5 additional analogs. The authors demonstrated that the compounds inhibited STAT3 binding to DNA, resulting in the suppression of non-small cell lung cancer and cisplatin resistant OvCa cell growth, suggesting potential therapeutic implications [218]. The compounds were found to inhibit STAT3 activity with Ki values ranging from 0.74–8.87 μM [218].

TTI-101 is a novel competitive STAT3 inhibitor developed by David Tweardy’s group in partnership with Tvardi Therapeutics [219]. The authors report that TTI-101 targets the Tyr705 site within the SH2 domain of STAT3, inhibiting STAT3 dimerization and phosphorylation [219]. TTI0101 was tested under GLP-compliant conditions for pharmacotoxicology profiles and was found to have no drug-related toxicity at the maximum dose administered [220]. Furthermore, the compound is currently under assessment in a Phase I clinical trial and is currently reporting no serious adverse events for dose level 4 [220]. In a study to assess TTI-101 effects on cellular function, it was determined that TTI-101 does not affect mitochondrial function or cause chemical modifications to STAT3, aggregation, or peripheral neuropathy [220]. Intriguingly, TTI-101 reduced nerve injury related to chemotherapy, demonstrating its potential use as an adjuvant therapy with peripheral neuropathy inducing chemotherapies [220].

### 6.3. STAT5

Pimozide was identified by Nelson et al. as a STAT5 inhibitor, observing that the compound suppressed STAT5 phosphorylation and induced apoptosis of AML cells [221,222]. More recently, pimozide has been shown to not be a selective STAT5 inhibitor, as it has also been shown to additionally suppress STAT3 phosphorylation [223,224]. Nevertheless, it has been demonstrated to suppress tumor growth and stemness properties in osteosarcoma [224]. In a recent study by Xiao et al., pimozide enhanced bromocriptine activity by suppressing STAT5-regulated cyclin D1 and Bcl-xL expression in prolactinomas [225]. 

Stafia-1 was developed by in silico screening of 10,369,180 compounds, with the original intention of identifying a novel STAT3 inhibitor [226]. Based on their screening workflow, the authors identified nine compounds from an O-phosphorylation virtual screen [226]. The parent scaffold used to develop Stafia-1 showed inhibition of STAT3 phosphorylation greater than 40% at 100 μM; however, when analyzed for specificity, it was determined to have greater activity towards STAT5a and STAT5b [226]. At this time, specific STAT5a inhibitors were lacking on the market, so chemical modifications were performed to optimize the compound for STAT5a, dubbed Stafia-1. The compound was determined to have a STAT5a Ki of 10.9 μM [226]. Moreover, STAT5b and STAT3 exhibited only 37% and 27% inhibition, respectively, at 200 μM concentrations, demonstrating the selectivity of Stafia-1 for STAT5a [226].

Stafib-1 and 2 were the first selective inhibitors specifically targeting the SH2 domain of STAT5b [227,228]. They were reported to bind to STAT5b with Ki values of 44 nM and 9 nM, respectively [228]. SH2 binding was confirmed by isothermal titration calorimetry and mutation analysis [228]. To test anti-cancer activity, Stafib-2 was developed into the prodrug Pomstafib-2. This also suppressed STAT5b phosphorylation and induced apoptosis of leukemia cells [228].

AC-4-130 was developed as a STAT5 inhibitor to improve upon Stafib-1 and 2 and [229]. AC-4-130 binds to the SH2 domain of STAT5, effectively inhibiting STAT5 phosphorylation and dimerization [229]. AC-4-130 significantly suppressed AML cells both in vitro and in vivo, demonstrating the relevance of targeting STAT5 [229]. Several recent studies have further evaluated AC-4-130 in lymphoma and AML, characterizing the role of STAT5 in maintenance of stemness, PDGFRβ-mediated oncogenic signaling, and FLT3 or TET2 mutated leukemias [230,231,232]. For example, Hadzijusufovic et al. determined that CD34+/CD38− myeloproliferative neoplastic (MPN) stem cells express high levels of phosphorylated STAT5 [232]. Treatment with AC-4-130-attenuated cell growth suggested that STAT5 played a critical role in MPN cell proliferation [232]. Seipel et al. investigated whether STAT5 inhibition could enhance the efficacy of FLT3 inhibitors to treat FLT3-mutated AML [211]. The authors evaluated AC-4-130 in combination with inhibitors of FLT3 (PKC412), BMI-1 (PTC596), MEK (trametinib), MCL1 (S63845), and BCL2 (venetoclax). The authors determined that AC-4-130 synergized with the S63845 in both FLT3 wild-type and mutated AML cells. Moreover, the presence of bone marrow stroma reduced the susceptibility of AML cells to all compounds except S63845 [230]. Collectively, the authors concluded that the combination of AC-4-130 with MCL1 inhibitors may serve as a novel treatment strategy [230]. 

### 6.4. STAT6

AS1517499 was reported as a selective STAT6 inhibitor by Chiba et al. in a 2009 study of IL-13-induced bronchial constriction [233]. Specifically, AS157499 inhibited IL-13-induced STAT6 phosphorylation at 100 nM, demonstrating potent activity [233]. In a murine model, IL-13 levels were significantly increased in response to an ovalbumin antigen challenge that resulted in the phosphorylation of STAT6 in bronchial tissues [233]. Pretreatment with 10 mg/kg AS157499 1 h before ovalbumin challenge significantly suppressed hypercontractility of bronchial tissues, IL-13 production, and STAT6 phosphorylation [233]. More recently, several studies have evaluated AS157499 in the treatment of asthma and immune regulation [234,235,236,237]. In the context of cancer, Binnemars-Postma et al. showed that STAT6 activation plays a critical role in M2 macrophage polarization [237]. Specifically, they determined that siRNA targeting STAT6 or using AS1517499 inhibited IL-4 and IL-13-induced M2 macrophage polarization in RAW264.7 cells [237]. Moreover, AS1517499 significantly reduced tumor growth and liver metastasis in a 4T1 mouse model of breast cancer [237]. This was accompanied by reduced F4/80 mRNA expression and M2 macrophage markers in tumor tissues, demonstrating that inhibition of STAT6 had potential therapeutic implications for cancer therapy [237]. In another study by Mendoza-Rodriguez et al., AS1517499 was evaluated as an adjuvant therapy for 5-FU in the treatment of colitis-associated colon cancers [238]. Specifically, the authors evaluated the anti-inflammatory agent, trimethylglycine, and AS1517499 in combination with 5-FU and determined that tumor growth was significantly reduced compared to 5-FU alone [238]. This was accompanied by decreased expression of STAT6 phosphorylation, markers of EMT, and pro-tumorigenic cytokines, IL-10, TGFβ, and IL-17 [238]. Collectively, these data indicate that STAT6 has potential for cancer therapy. 

Leflunomide is one of two FDA approved therapies targeting STAT6. Siemasko et al. showed in 1998 that leflunomide suppressed JAK3 and STAT6 phosphorylation [239]. It has since been shown to modulate de novo pyrimidine synthesis as well as to inhibit S6K1 [240,241,242]. Nevertheless, several studies have demonstrated anti-cancer activity of leflunomide through the suppression of STAT6 signaling [243,244,245]. 

Vorinostat is the second of two FDA approved therapies targeting STAT6. Buglio et al. showed that vorinostat inhibited STAT6 phosphorylation and transcription in Hodgkin lymphoma, resulting in G2/M cell cycle arrest mediated by p21 [246]. Importantly, it has recently been extensively characterized as an inhibitor for both class I and class II HDACs, and a DNA-damaging agent [247,248,249], indicating STAT6 independent effects. 

TMC-264 is a STAT6 inhibitor developed from the fermentation broth of Phoma sp. TC 1674 [250]. Initial studies determined that TMC-264 suppressed STAT6 phosphorylation at IC50 values of 1.6 μM, while STAT5 phosphorylation was suppressed with an IC50 value of 16 μM, and STAT1 phosphorylation was unaffected [250]. In the context of cancer, Lai et al., determined the bioactive profile of several dibenzo-α-pyrone derivatives from the fungus, Rhizopycnis vagum Nitaf22 [251]. Several compounds demonstrated antibacterial activity and TMC-264 was the only compound to demonstrate cytotoxicity towards five human cancer cell lines, which included HCT116, Hep62, BGC-823, NCI-H1650, and A2780 [251]. IC50 values ranged from 3.2 to 7.8 μM [251]. These studies did not evaluate the effect on STAT6 phosphorylation, so further studies are warranted to characterize the role of STAT6 in the TMC-264-mediated cytotoxicity of cancer cells.

## 7. Conclusions and Future Directions

Ovarian cancer still presents numerous challenges, requiring continued research due to its prevalence and high rate of mortality. As outlined in the review above, STAT proteins play an important role in tumor growth, metastasis, stemness, angiogenesis, and chemoresistance. What is also evident is the fact that of the STATs, the best studied and targeted factors are STAT3 and STAT5. In recent years, the development of selective small-molecule inhibitors, summarized in Table 2, targeting STAT3 and STAT5 have been developed and, specifically, STAT3 inhibitors such as TTI-101 are in early-phase clinical trials to assess safety and toxicity [220]. It is important to follow these studies and evaluate these compounds in STAT-dependent malignancies. More importantly, it is clear that other STAT family members—STAT1, STAT2, STAT4, and STAT6—also contribute critical roles to cancer progression, either modulating the cancer cell directly or by altering cells of the TME. There remain prominent gaps in our knowledge related to the molecular function of these STATs in OvCa and other malignancies. It is imperative that further research is performed to narrow this knowledge gap. Moreover, due to the deficiency in our understanding of STAT1, STAT2, STAT4, and STAT6 in cancer, selective inhibitors also remain elusive. This provides a significant opportunity. Currently, based on our literature search, there are no selective inhibitors for STAT2 or STAT4, and yet STAT2 and STAT4 enhance cancer progression by promoting EMT, [7,94]. In addition to targeting the cancer cell, it is becoming apparent that focusing on the cells in the TME may also pay dividends. An example of this is the STAT6 inhibitor, AS1517499, which has been shown to inhibit M2 macrophage polarization, thereby reducing breast cancer growth and metastasis [237]. This clearly justified the use of STAT inhibitors in an adjuvant setting to manipulate the TME and thereby enhance the therapeutic efficacy of standard-of-care agents. Future studies could therefore focus on combination therapy with such agents. 

In summary, STATs are an attractive target for therapeutic development due to their constitutive activation and overexpression in numerous cancer types, which drives pro-tumorigenic signaling cascades. Further elucidating the role of each STAT in cancers and in the TME, especially in CAFs, TAMs, and other immune cells, is critical to improving therapy. We have only begun to scratch the surface of STAT biology; there remains ample opportunity to progress our understanding of the regulation of gene expression and the stability and activation of these proteins. Finally, there is a need to focus on drug development, particularly with respect to STAT2 and STAT4, and to assess lead candidates through clinical trials.

## Figures and Tables

**Table 1:** Activators of STAT signaling.

Proteins	Activators	Biologic Response	References
STAT1	IFNα, IFNβ, IFNγ, IFNλ, EGF, PDGF, IL6, IL27	Promote inflammation, PD-L1 expression, apoptosis, MYC gene expression, monocyte activation	[45,46,47,48,49]
STAT2	IFNα, IFNβ, IFNλ	Promote inflammation	[50]
STAT3	IL6, IL6 family cytokines, IL10, IL22, Prolactin, Growth Hormone, EGF, TGFα, VEGF	Promote proliferation, chemoresistance, cell migration	[51]
STAT4	IFNγ, IL12, IL23, IL2, IL35	Promote T Cell differentiation, IFNγ production, NK cell activation	[52]
STAT5	Growth hormone, Prolactin, IFNα, IFNβ, IL3, Thrombopoietin, Erythropoietin, GM-CSF	Promote proliferation, cell migration	[53,54]
STAT6	IL4, IL13	Promote macrophage polarization, EMT	[55,56]

**Table 2:** Inhibitors of STATs.

Protein	Inhibitors	Interacting Site	References
STAT1	Fludarabine	N/A	[252]
	ISS840	STAT1 SH2 domain	[145]
	Pravastatin	N/A	[146]
	THIF	STAT1 SH2 domain	[150]
STAT2	N/A	N/A	N/A
STAT3	Indirubin	C-Src, CDK, GSK3β, STAT3 DB domain	[152,154]
	Resveratrol	Src	[155]
	Curcumin	STAT3 CC domain	[253]
	FLL31/32	JAK2, STAT3 SH2 domain	[175]
	HO-3867/4200	STAT3 DB domain	[175]
	H-4318	STAT3 DB domain	[176]
	Corosolic acid	N/A	[254]
	Cucurbitacin -I, -B, -E	JAK2, STAT3	[183,184,185]
	323-1/323-2	STAT3 SH2 domain	[191]
	Stattic	STAT3 SH2 domain	[192]
	STA-21	STAT3 DB domain	[200]
	LLL-3/LLL-12	STAT3 SH2 domain	[201]
	LC28	STAT3 SH2 domain	[218]
	TTI-101	STAT3 SH2 domain	[220,255]
STAT4	N/A	N/A	N/A
STAT5	Pimozide	N/A	[221]
	Stafia-1	STAT5a SH2 domain	[226]
	Stafib-1/2	STAT5b SH2 domain	[227,228]
	AC-4-130	STAT5 SH2 domain	[229]
STAT6	AS1517499	N/A	[256]
	Leflunomide	STAT6 DB domain	[239]
	Vorinostat	N/A	[246]
	TMC-264	STAT6 DB domain	[250]
